# Supporting Medication Adherence in Pediatric Patients Undergoing Hematopoietic Stem Cell Transplant Using the BMT4me mHealth App: Mixed Methods Usability Study

**DOI:** 10.2196/66847

**Published:** 2025-05-29

**Authors:** Mariam Kochashvili, Parishma Guttoo, Emre Sezgin, Ahna Pai, Rajinder Bajwa, Wendy Landier, Cynthia Gerhardt, Micah Skeens

**Affiliations:** 1 Center for Biobehavioral Health, The Abigail Wexner Research Institute, Nationwide Children's Hospital, 431 South 18th Street, Columbus, OH, 43205, United States, 1 6147228958; 2 The Ohio State University, Columbus, OH, United States; 3 Nationwide Children's Hospital, Columbus, OH, United States; 4 Division of Pediatric Hematology Oncology, University of Alabama at Birmingham, Birmingham, AL, United States

**Keywords:** mHealth, pediatric transplant, digital health, medication adherence, usability, hematopoietic stem cell transplant, bone marrow transplant, pediatric, children, hematopoietic stem cell, HSC, smartphone, mobile health, BMT4me, digital health intervention, descriptive statistics, thematic analysis, usability study, mixed method, social support, health outcomes, medication management, symptom tracking, electronic medical records, user-friendly

## Abstract

**Background:**

Due to multifaceted outpatient regimens, children receiving hematopoietic stem cell transplants (HCTs) are at high risk of medication nonadherence, leading to life-threatening complications. Mobile health (mHealth) interventions have proven effective in improving adherence in various pediatric conditions; however, adherence intervention literature on HCT is limited.

**Objective:**

This study aimed to assess the usability of a mHealth intervention (BMT4me) designed to serve as a real-time, personalized tool for medication management or adherence, symptom tracking, and journal keeping.

**Methods:**

Following a mixed methods approach, 14 caregivers (n=11, 79% female; n=10, 71% White) of children aged 2‐18 (mean age 8.51, SD 5.18) years in the acute phase (first 100 d) post-HCT were recruited. Caregivers were asked to use the BMT4me app for 100 days or until weaning of the immunosuppressant medications to measure usability. The System Usability Scale (assessing functionality and acceptability), reaction cards (assessing desirability), caregiver satisfaction (assessing satisfaction) with the app, and semistructured interviews (assessing participant experience using the app and feedback regarding features) were conducted at two time points, at enrollment and study completion.

**Results:**

The mean System Usability Scale score was 86.15 (SD 12.81) at enrollment and 73.13 (SD 16.13) at study completion, with most participants reporting the app easy to use and accepable during both time points. At enrollment, 80% (n=12) of caregivers reported that the app was effective in motivating them to stay on schedule, and 87% (n=13) indicated they would recommend it to others. At study completion, 75% (n=6) of caregivers found the app helpful for tracking their child’s medication schedule, and 64% (n=5) would recommend it to others. Caregivers described the app as “accessible,” “useful,” and “valuable.” Qualitative interviews during both time points revealed caregivers’ positive reactions to the app, particularly regarding medication reminders, tracking symptoms, and notes features, while also providing suggestions for improvements, such as integrating the BMT4me app with electronic medical records, incorporating educational content, adding fields for recording vital signs, and important phone numbers.

**Conclusions:**

The BMT4me app demonstrated promising usability as a mHealth intervention among pediatric patients undergoing HCT. Caregivers considered the app user-friendly and valuable, with positive feedback on its features, such as medication reminders and symptom tracking. Despite minor reported issues with app functionality, the overall acceptance of the app suggests its potential to support families in managing complex treatment. The findings from this study will inform the feasibility of testing in larger randomized controlled trials.

## Introduction

Pediatric hematopoietic stem cell transplants (HCTs) are an intensive life-saving treatment for several malignant and nonmalignant disorders [1]. However, HCT often requires a long hospital stay that is stressful for the children and their caregivers [2]. Symptom and medication management are important components of the HCT experience, and it is critical for caregivers to adhere to recommendations and communicate with the care team [3]. After discharge, caregivers must follow complex medication regimens with various dosages and frequent dose adjustments, increasing the risk for nonadherence [4,5]. In the pediatric HCT population, 52% to 73% [2,6,7] of patients do not take medications as they are prescribed during the treatment course. Therefore, medication adherence is a primary concern for health care providers and caregivers after pediatric HCT [8,9]. Many factors impact medication adherence rates, including patient-related factors, forgetfulness, therapy side-effects, complexity and length of treatment, and route of administration [10-13]. Although medication adherence in pediatric HCT is understudied and interventions are limited, research in other pediatric chronic conditions has demonstrated the potential of mobile health (mHealth) interventions in improving medication adherence [14-16].

As smartphones become nearly ubiquitous in daily life, mHealth interventions can improve families’ ability to manage their child’s medical care [17,18]. Recent estimates show that over 5 billion people have access to mobile phone services around the world [18]. Additionally, a study of adults with chronic diseases suggests that mobile apps as mHealth intervention tools are more effective for improving medication adherence than non-mHealth interventions [19]. mHealth interventions have resulted in better clinical outcomes (eg, increase in health-related quality of life, symptom management, and decrease in readmissions and treatment anxiety) [20-22] through behavior change and enhancement of adherence to treatment [23-25]. mHealth interventions allow individuals and caregivers to track medication doses and symptoms, make notes of discussion points with their health care team, and find educational resources and support networks [26-29]. However, such interventions have yet to be tested to promote medication adherence among children in the acute phase post-HCT (ie, hospital discharge to day 100) [30].

This paper reports a longitudinal mixed methods pilot study examining the usability of a mHealth intervention (BMT4me) with caregivers of children in the acute phase following HCT. This intervention helps caregivers to record and track their child’s medications, set reminders, report symptoms, and take notes on their child’s progress. The goal is to inform future refinement of the intervention, a feasibility trial, and a pilot randomized controlled trial examining efficacy.

## Methods

### Study Design

Data are from a longitudinal mixed methods study to assess the usability of a newly developed mHealth intervention for pediatric post-HCT medication management. The study was conducted at a large Midwestern children’s hospital from September 2021 to January 2023. Eligible caregivers were identified from the HCT clinic schedule and inpatient HCT unit based on the following eligibility criteria: (1) English-speaking, (2) 18 years of age or older, (3) having a child between 2 and 18 years of age undergoing allogeneic HCT, and (4) having a smartphone (either Android or iPhone) at recruitment and during the study period. All 20 caregivers of children who received HCT during the study period and met the eligibility criteria were approached for participation. A total of 15 caregivers consented to participate in the study, while 5 caregivers declined due to reasons including being busy with caring for the child and not being comfortable using the apps in general. One caregiver withdrew after initial consenting, resulting in a final cohort of 14 caregivers.

### Ethical Considerations

The study was approved by the Nationwide Children’s Hospital Institutional Review Board (approval STUDY00000910) and was designed in accordance with the ethical standards laid out by the Declaration of Helsinki. Eligible interested participants provided informed consent prior to enrollment and were assigned ID numbers for confidentiality. Recordings of qualitative interviews were destroyed after completion of the transcription, and identifying information in the transcripts was removed. All participant information has been anonymized in this paper, including the text, tables, and figures. Upon completion of the study, all participants received a US $20 gift card as compensation.

### Measures

#### Demographic Data Form

Caregivers self-reported the child’s and their own background characteristics, including age, sex, race, ethnicity, education level, and family income at the time of study enrollment.

#### System Usability Scale

Caregivers rated 10 items on a 5-point Likert scale to evaluate the functionality and acceptability of the BMT4me app. Total scores range from 0 to 100, with scores >68% considered above average [31]. Internal consistency (Cronbach α=0.91) and convergent validity (*r=*0.81 with a 7-point scale of “user-friendliness”) have been well established [32,33].

#### Caregiver Satisfaction

Investigators developed a 9-item survey to obtain feedback regarding caregiver app use, benefits, burdens, barriers, suggested modifications, and overall satisfaction. Questions were rated on a 1 to 4 scale, with higher total scores indicating greater caregiver satisfaction.

#### Reaction Card

Reaction cards were developed by Microsoft as part of a “desirability toolkit” to elicit immediate reactions, thoughts, or opinions from individuals regarding a particular tool or technology [34]. Using a reaction card of 55 listed words, caregivers provided feedback on the desirability of the BMT4me app. The words and the number of times they were chosen were summarized to indicate the overall attitude toward the app. Higher frequencies of positive words indicated greater usability, while higher frequencies of negative words indicated lower usability [35].

#### Qualitative Interview

Qualitative nterview guides were developed using a combination of literature review, expert consultation, and pilot testing to ensure relevance and clarity. Questions were designed to elicit in-depth perspectives on key study themes while allowing for flexibility in participant responses. A semistructured format was used to balance consistency across interviews, with the opportunity for participants to elaborate. The semistructured interviews were transcribed verbatim and analyzed using NVivo software (QSR International).

#### BMT4me App

**Figure 1.**
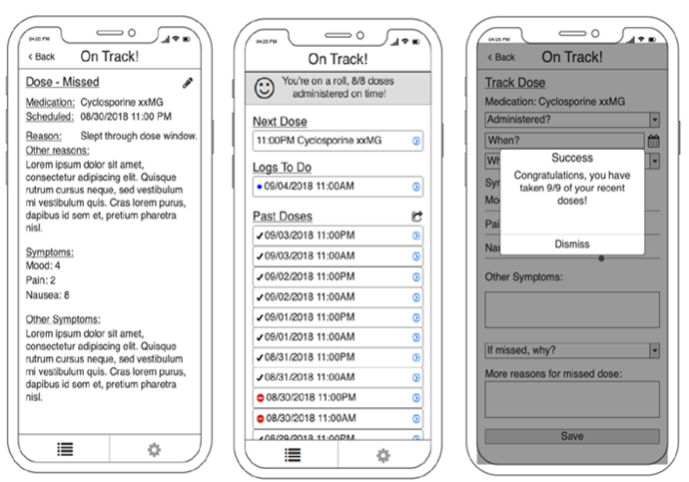
Version 1 of mobile health app wireframes.

The development of the BMT4me app (Figures1  2) followed a user-centered, multiphase iterative approach. This method actively involved patients, caregivers, and health care providers at every stage of the app’s creation. Initially, a wireframe (Figure 1) was designed, serving as a simple visual representation of the app’s structure and content [36].

**Figure 2.**
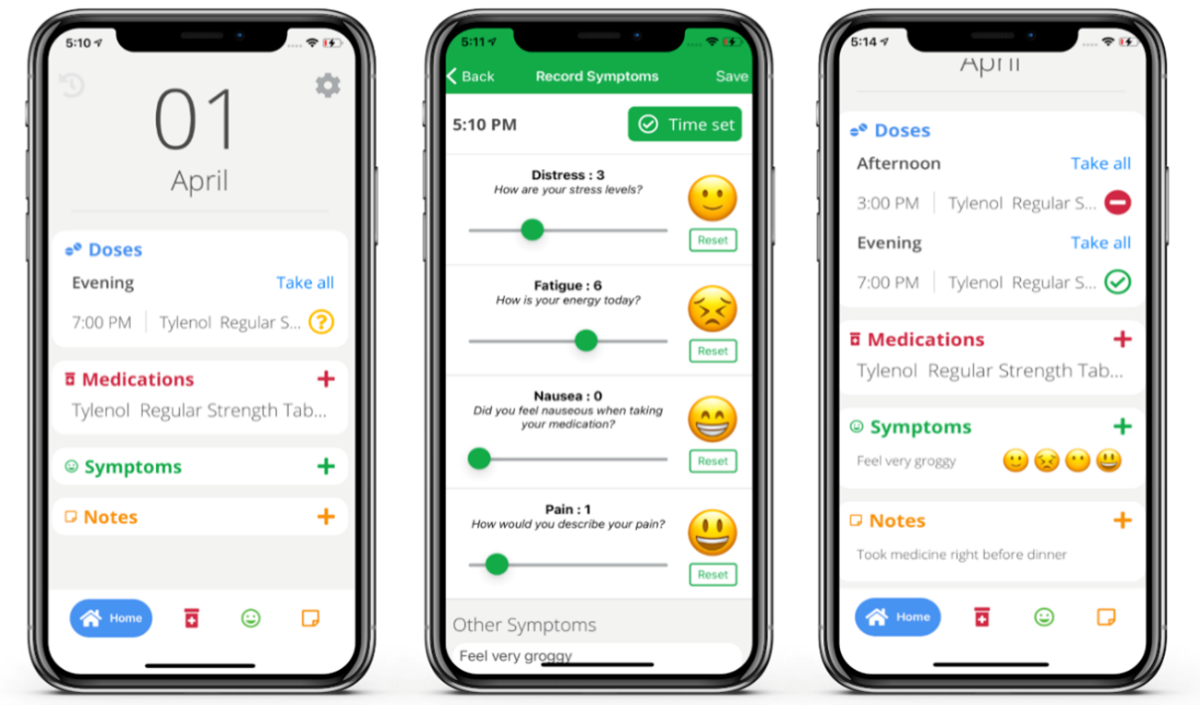
Revised BMT4me app prototype.

Feedback was then collected from caregiver and child dyads, which informed the creation of the BMT4me app prototype (Figure 2). Afterward, health care providers assessed the prototype, and feedback led to further refinement of the app.

The BMT4me app was developed for both iOS and Android devices as a real-time, personalized medication management or adherence tool, and to track medication, symptoms, and side effects. Medications, doses, and schedules can be manually typed into the app by the user or entered using the image-to-text feature, which converts the medication label into text. Through pop-up notifications, the BMT4me app reminds caregivers of the medication doses and schedule, while also recording the time medication was taken and reasons for missed doses. Symptom ratings are represented by emojis on a slider scale of 1 to 10. Additional features include a note section to upload pictures and for recording any details of care to communicate with the provider. In the case of shared caregiver responsibility, the app can be installed on separate devices using the same sign-in code to sync information in real-time between caregivers. Amazon Web Services were used for data storage and app database management.

### Procedures

If eligible, recruitment occurred either before or on the day of the child’s discharge following HCT or at the first HCT clinic visit postdischarge. Trained research staff introduced the study, and interested caregivers provided written informed consent prior to participation.

To facilitate app login and use, participants received a unique QR code for activating the BMT4me app on their smartphones, and the research team helped with installation. Understanding and engagement with the app were evaluated in the following steps (further explained in [37]).

#### Step 1: Unobtrusive Observation to Measure the Intuitiveness of the Interface (10‐15 Min)

##### Ease of Navigation

This was how quickly and accurately participants were able to find features and complete tasks, as well as any moments of hesitation or confusion when navigating the menu and buttons.

##### Task completion

This was how quickly and accurately participants found features or completed tasks such as inputting medications, symptoms, or notes.

##### Error frequency

This included mistakes made while interacting with the app, such as tapping the wrong button or misunderstanding the instructions.

##### Flow and Progression

This was how naturally participants moved through the app interface without guidance.

Caregivers were encouraged to interact with the app independently, without guidance from the study staff, to evaluate the intuitiveness of the app’s interface. During this phase, research staff observed and recorded participant progress related to the other steps described below.

### Step 2: Interactive Observation (10‐15 Min)

After observing independent app use, the research team started to interrupt the participants and ask questions regarding observed cues. Caregivers were encouraged to share their thoughts and criticisms of the app. The discussion was based on individual participant cues, and preprescribed questions were not possible.

### Step 3: Debriefing (15‐20 Min)

Caregivers were asked to share their experience with the app, including the app’s interface and content, as well as thoughts on incorporating the app into their daily routine. Feedback regarding the strengths and weaknesses of the app was also collected.

### Step 4: Passive Use Observation

To ensure caregivers understood the app, research staff provided a detailed introduction, answered participant questions, and had the caregivers practice adding medications and tracking symptoms. Participants were invited to continue using the BMT4me app at home for 100 days or until the child had been weaned off the immunosuppressant medications. Participants’ passive use data on app use and phone activity was digitally logged

Caregivers then evaluated the usability of the BMT4me app at the beginning and end of the study, using the System Usability Scale (SUS), Caregiver Satisfaction, and Reaction Card assessments. SUS, Caregiver Satisfaction, and Reaction cards were completed after participants independently interacted with the app for 5‐10 minutes during enrollment. Participants completed enrollment measures in the hospital prior to discharge, and they had an option to complete them either electronically via REDCap (Research Electronic Data Capture; Vanderbilt University) on a study iPad or using paper and pencil. Exit measures were completed electronically, where participants were emailed the survey link. Participants were invited to continue using the BMT4me app at home in the acute phase post-HCT (100 d or until the child had been weaned off the immunosuppressant medications) because the risk of nonadherence and complications such as GVHD is highest during that time. The BMT4me app collected daily data on medication-taking, the time medication was taken, reasons for missed doses and barriers, symptoms, and notes. Passive use data on phone activity and app use were digitally recorded and sent to the research team by software developers each week. During the study, research staff followed up with participants weekly to provide technical app support if needed.

Upon completion of surveys, semistructured interviews were also conducted in-person, both at the beginning and end of the study, to explore app use experiences, helpful features, suggestions for future improvements, and barriers encountered while using the app at home. To schedule the interviews, participants were contacted by research staff via phone call or email. During their child’s clinic visit, semistructured interviews, lasting approximately 15‐20 minutes, were conducted by research staff trained in qualitative interview methods. The study investigator conducted fidelity checks to ensure consistency across the research staff’s qualitative interview techniques. All interviews were audio-recorded for analysis.

### Data Management and Analysis

Data cleaning and verification were completed using Excel (Microsoft Corp), and the data were then analyzed using SPSS software (version 26; IBM Corp). Descriptive statistics (frequencies, means, and SDs) summarized quantitative data, including demographic characteristics, BMT4me app activity, and survey responses. During the passive observation period, passive data modules recorded phone activity and caregivers’ app use (such as time, date, and duration of use). Descriptive statistics were applied to analyze the phone activity, and correlation analysis was conducted to examine use patterns over time. Usability and acceptability of the BMT4me app were assessed by averaging total scores from the SUS. The proportion of participants who enrolled and completed the study was examined to assess the feasibility of the intervention.

Semistructured interviews were transcribed verbatim for content analysis and were organized and coded using NVivo software. Initially, the study team read the transcripts to familiarize themselves with the data. Afterward, the team generated an initial list of codes to align with study questions, and later, the codes were sorted to create a thematic framework. For consistency and accuracy, themes and code groups were then revised systematically by the team’s identified coders (MS, MK, and Kathryn A Vannatta), and the thematic framework was adjusted to reflect any changes. Once the review was complete, final codes and themes were reviewed by the study’s principal investigator (MS), and any disagreements were resolved through consensus with the coding team. Finally, findings were interpreted and reported in relation to existing literature.

## Results

### Participants

Initially, 15 caregivers were enrolled in the study (Table 1). Among them, 10 (67%) were approached at discharge and 5 (33%) at the first BMT follow-up visit postdischarge. One caregiver opted out after consenting for the study, and 2 were lost to follow-up, one at week 2 and the other at week 3, both due to transfer of care to another institution. Most caregivers were female (n=11, 79%), White (n=10, 71%), non-Hispanic (n=13, 93%), married (n=9, 64%), and had a college-level education (n=8, 57%). More than half of caregivers reported an annual family income of ≤US $75,000 (n=8, 57%). All caregivers were biological parents of the children. The sample of children was mostly male (n=10, 71%), White (n=10, 71%), and non-Hispanic (n=13, 93%).

**Table 1.** Caregiver and child demographic characteristics.

Characteristics	Value (n=14)
Caregiver demographic characteristics	
Caregiver age, years	
Mean (SD)	37.92 (7.62)
Median (IQR)	39.0 (25‐43)
Sex, n (%)	
Male	3 (21)
Female	11 (79)
Marital status, n (%)	
Single	1 (7)
Married	9 (64)
Divorced	1 (7)
Separated	3 (21)
Highest grade of school completed, n (%)	
College	8 (57)
High school	2 (14)
Graduate or professional	1 (7)
Post-secondary high school (technical or trade school)	2 (14)
Not reported	1 (7)
Annual family income (US $), n (%)	
Under 25,00	3 (21)
25,001-50,000 per year	4 (29)
50,001-75,000 per year	1 (7)
75,001-100,000 per year	2 (14)
100,001-150,000 per year	2 (14)
150,001 or more	1 (7)
Not reported	1 (7)
Caregiver’s race, n (%)	
Asian	1 (7)
Black or African American	3 (21)
White	10 (71)
Caregiver’s ethnicity, n (%)	
Hispanic or Latin	1 (7)
Not Hispanic	13 (93)
Caregiver’s device used, n (%)	
iOS mobile phone	11 (79)
Android mobile phone	3 (21)
Child demographic characteristics	
Child age (years)	
Mean (SD)	8.51 (5.19)
Median (IQR)	7.98 (2.01‐11.36)
Child’s sex, n (%)	
Male	10 (71)
Female	4 (29)
Child’s race, n (%)	
Asian	1 (7)
Black or African American	2 (14)
Other, please specify	1 (7)
White	10 (71)
Child’s ethnicity, n (%)	
Non-Hispanic	13 (93)
Not reported	1 (7)
Child education, n (%)	
Preschool	1 (7)
Grade 1	2 (14)
Grade 3	1 (7)
Grade 4	3 (21)
Grade 10	2 (14)
Graduated high school	1 (7)
Not reported	4 (29)

### Usability

#### SUS

**Figure 3.**
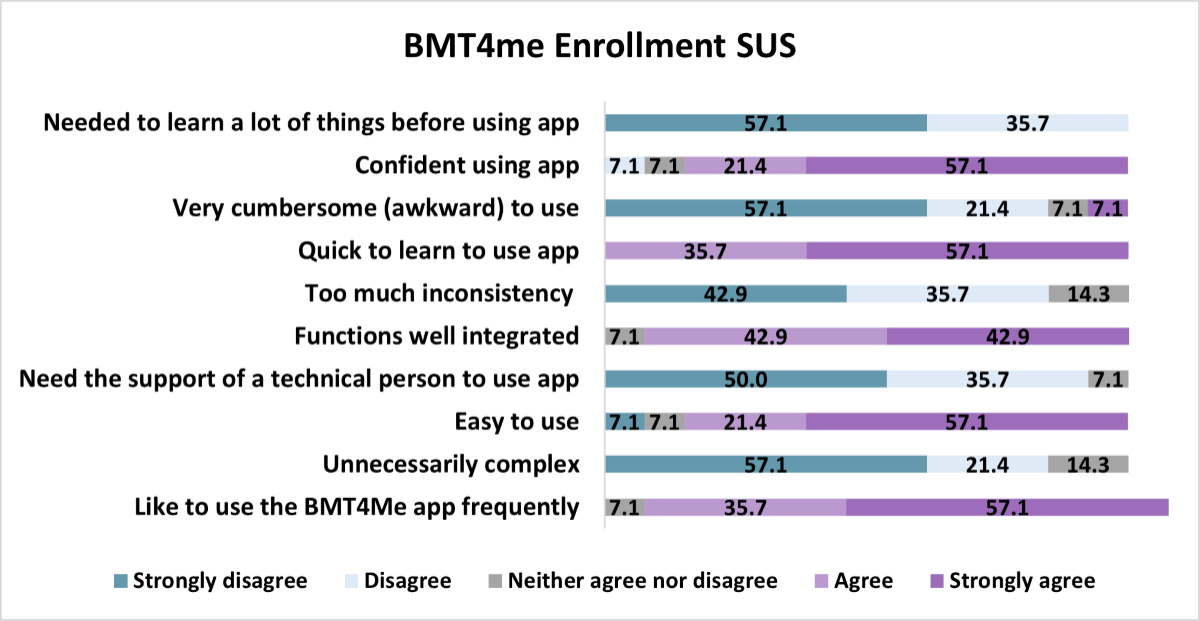
Enrollment SUS survey responses from caregivers. SUS: System Usability Scale.

At enrollment, the average SUS score was 86.15 (SD 12.81), and the median was 87.5 (IQR 62.5‐97.5). On individual items, caregivers reported that the app was quick to learn (n=13, 93%), they were confident using the app (n=11, 79%), the app was easy to use (n=11, 79%) and that they would like to use the BMT4me app (n=13, 93%; Figure 3).

#### Caregiver Satisfaction

**Figure 4.**
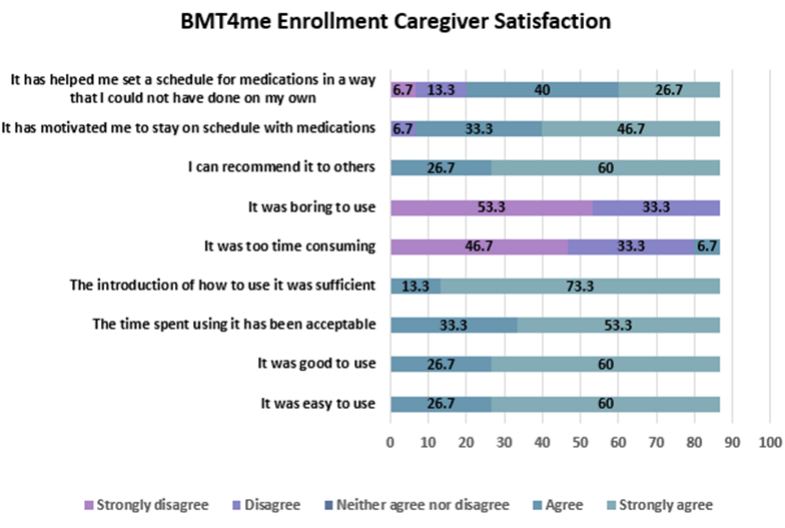
Enrollment caregiver satisfaction survey responses.

Participants reported that the BMT4me app was easy to use (n=13, 87%) and effective in motivating them to stay on schedule with medications (n=12, 80%). Most did not find it time-consuming (n=12, 80%) or boring (n=13, 87%). Caregivers indicated they would recommend the app to others (n=13, 87%) and felt it helped them maintain their child’s medication schedule in ways they could not have managed on their own (n=10, 67%; Figure 4).

#### Reaction Cards

Most caregivers expressed a positive reaction after their initial use of the app with the top three endorsed reactions being the app was accessible (n=11, 73%), useful (n=11, 73%), and easy to use (n=10, 67%), which received the highest percentage of responses (Multimedia Appendix 1).

#### SUS

**Figure 5.**
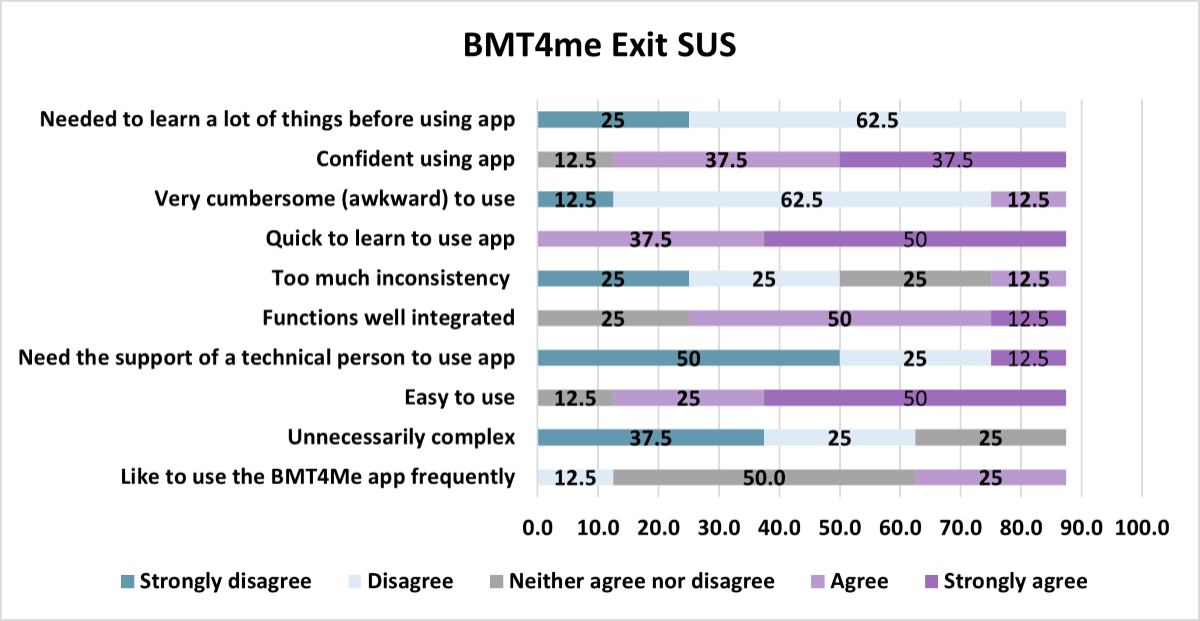
Exit SUS responses from caregivers. SUS: System Usability Scale.

At study completion, the mean SUS score was 73.13 (SD 16.13), and the median was 75.0(IQR 50‐86.3). On individual items, caregivers reported the app was quick to learn (n=7, 88%), they were confident in using the app (n=6, 75%), app was easy to use (n=6, 75%), and that features were well-integrated (n=5, 65%; Figure 5).

#### Caregiver Satisfaction

**Figure 6.**
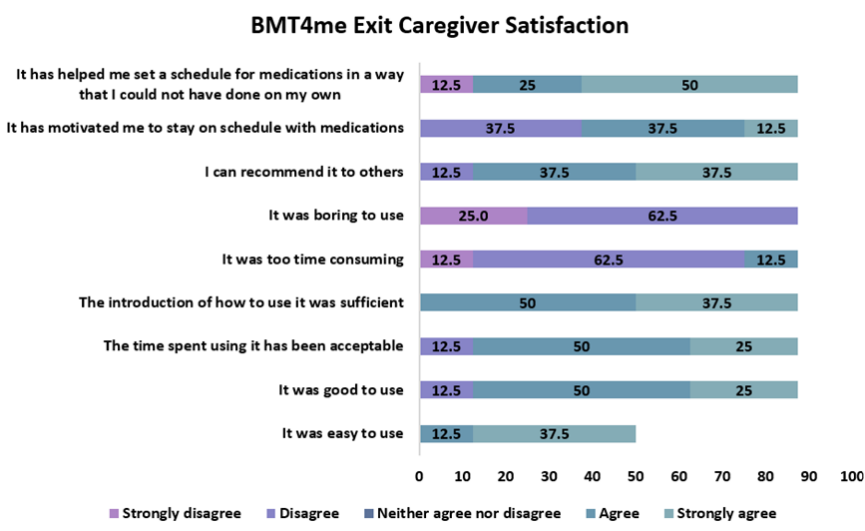
Exit caregiver satisfaction survey responses.

Caregivers reported that the app was helpful in keeping track of their child’s medication schedule (n=6, 75%), while also being good to use (n=6, 75%) and not boring (n=7, 88%). A total of 6 caregivers indicated that they would recommend the app to others (n=6, 75%; Figure 6).

#### Reaction Cards

After a few weeks of interaction with the app at home, most caregivers expressed positive reactions to the app. The top three endorsed reactions were accessible (n=7, 88%), useful (n=7, 88%), and valuable (n=7, 88%), which received the highest percentage of responses (Multimedia Appendix 2).

#### Feasibility

**Figure 7.**
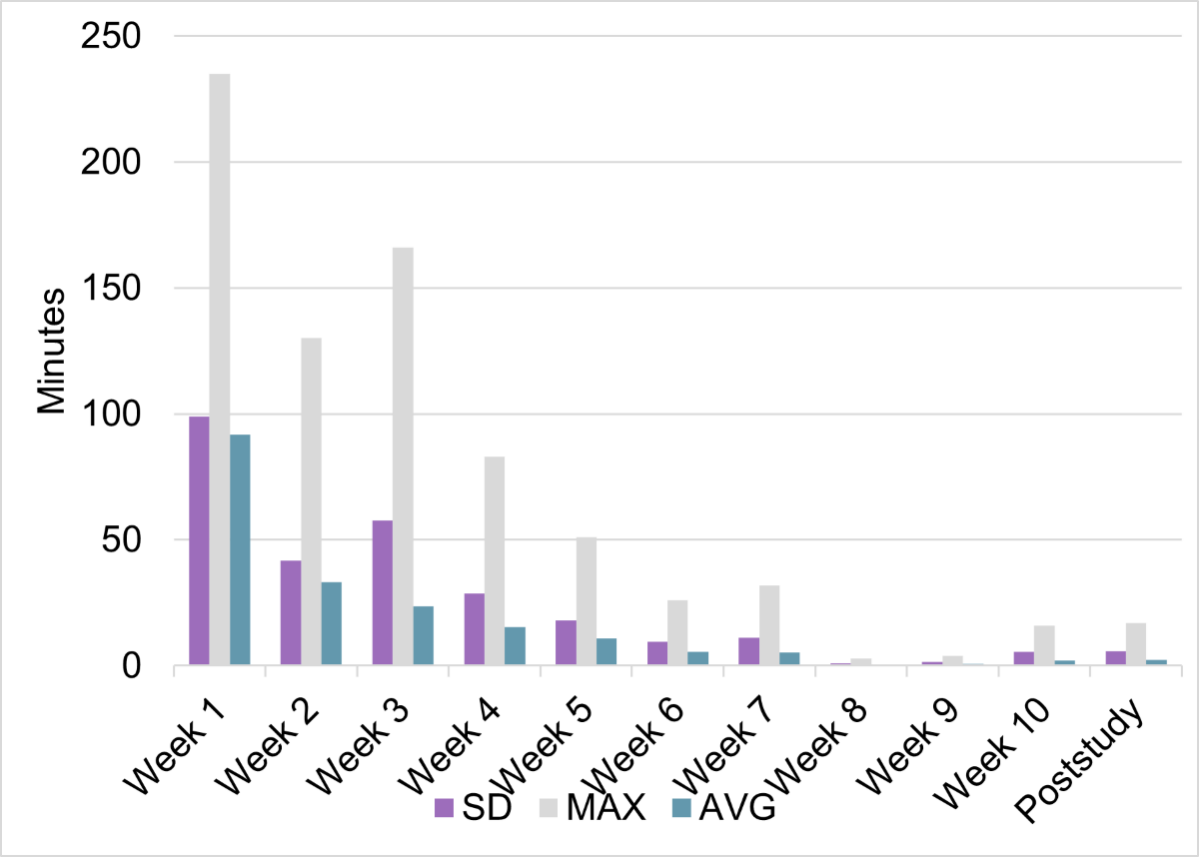
Weekly app engagement.

Of the 14 caregivers who successfully installed the app, 7 (50%) caregivers did not use the app after the initial sign-in, 2 (14%) caregivers added their child’s regimen, and 5 (36%) caregivers used the app for at least 1 week. In total, 2 (14%) caregivers used the app for 1 week, while the other 3 (21%) caregivers used the app until study weeks 2, 6, and 7, respectively (Figures7  8).

Descriptive analyses were conducted for the app data (Multimedia Appendix 3) . A total of 1579 app engagement activities were recorded, of which 286 (18%) were for “loading the app,” suggesting the number of times enrolled caregivers either attempted logging in or opening the app. The “creating a medication” feature, indicating medication entry into the app, was used 172 (11%) times. The feature “create a dose” was used 552 (35%) times, reflecting the number of times a medication was either entered or edited. A total of 100 (6%) responses were registered for the “take-all-doses.” The “take-all-doses” feature pertained to the number of times enrolled caregivers registered on the app that their child took all their prescribed medications. The note feature was used 24 (2%) times. Caregivers used the app to enter their child’s symptoms 22 (1%) times. Four of the enrolled caregivers used the note feature at least once.

**Figure 8.**
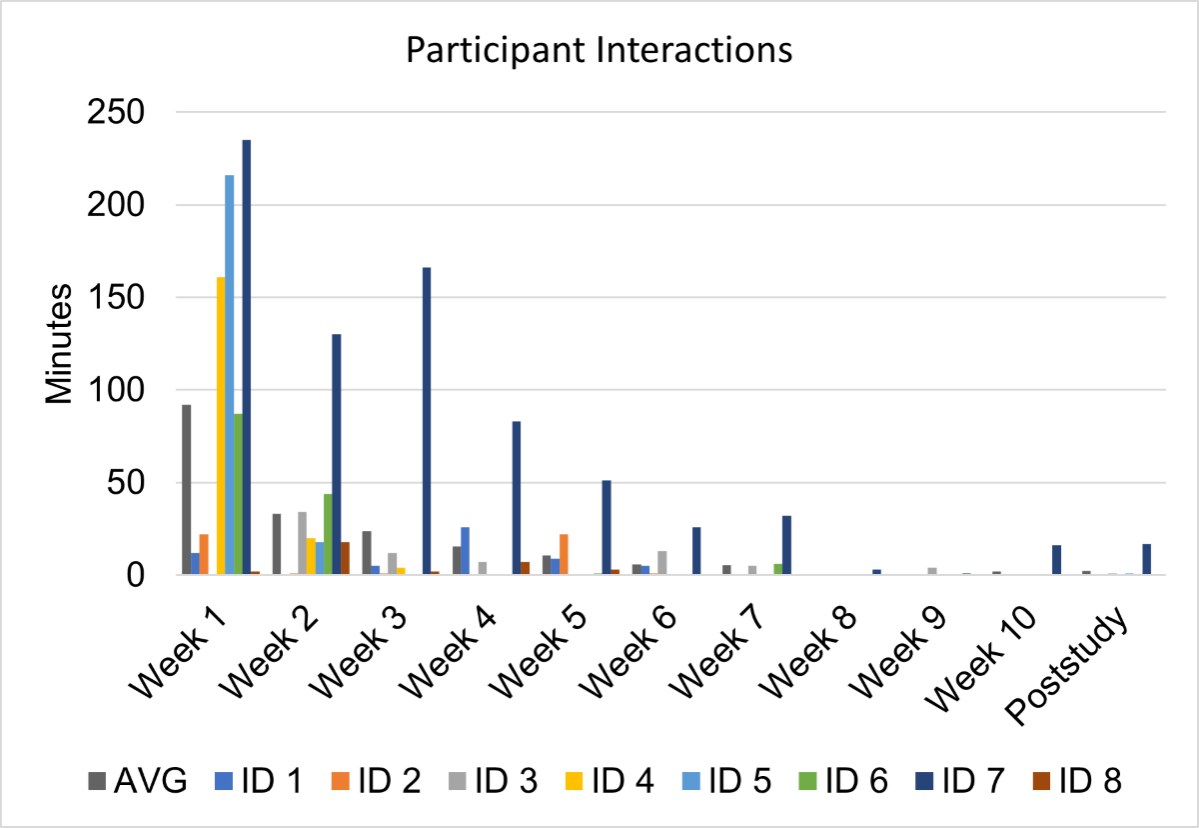
Weekly app engagement by individual user.

### Semistructured Interviews

Our qualitative analysis of both enrollment and exit semistructured interviews found that medication reminders were highly favored by 43% (n=6) of families, while recording side effects and symptoms were reported by 21% (n=3) of participants, and writing notes was favored by 14% (n=2). Participants also suggested several new features for the app (Table 2). Half of the families (n=7, 50%) recommended integrating the app with electronic medical record (EMR) to automatically add prescribed medications and appointments and to allow providers to review app activity for pattern identification. EMR is a secure member website that allows patients to access their health information, view appointments, medical test results, and medication therapies, and communicate with their providers [38]. Additionally, 14% (n=2) of families suggested including educational content about common symptoms posttransplant and a search field for medications and their uses. Another 14% (n=2) recommended a field for recording vital signs, such as temperature and blood pressure. Finally, 14% (n=2) proposed adding important contact numbers such as pharmacies, providers, and support groups to the app.

**Table 2.** Features suggested by participants.

Features families suggested	Value, n (%)
Integrate with EMR^a^ to automatically add prescribed medications and appointments to the app, as well as allowing the provider to review app activity to identify patterns.	7 (50)
Including educational content such as frequently experienced symptoms after transplant, and a field to look up medications and for what they are used.	2 (14)
A field for recording vitals such as fever and blood pressure on the app.	2 (14)
Include important numbers in the app, such as pharmacy, provider, and support groups.	2 (14)

a EMR: electronic medical record.

During the enrollment interviews, 3 key themes regarding usability and features of the app were identified (Table 3). Ease of use was the most frequently recurring theme. Participants repeatedly highlighted the intuitive design, which made navigation and operation simple, especially for those who were less technologically skilled. Several participants remarked that grandparents, who lack digital proficiency, could use the app without any issues. Another significant theme was the app’s ability to keep families “on track” with medication schedules and doses. Caregivers expressed enthusiasm for using the app at home to manage a variety of medications and doses and record symptoms. Participants appreciated the ability to mark off administered medications and track notes, finding the digital logging of all information in one place helpful.

**Table 3.** Participants' reflections on the use of the BMT4me app.

Themes identified from qualitative interviews	Quotes from enrollment interviews	Quotes from exit interviews
Easy to use	“It’s very simple. Even if my mom had to do that, I think it would be simple for her to use it.” [ID 5] “It looks straightforward and easy to use.” [ID 3]	“It was pretty easy to use. It’s pretty intuitive, I think. I think it’s easy to pick it up and just start entering things, which is very helpful. Like, it’s pretty straightforward.” [ID 3] “It’s really pretty self-explanatory. So easy usage which is always nice because if it’s complicated, you kind of don’t want to deal with it, you know, human nature kind of thing.” [ID 11]
Keep us on track	“I think it’d be useful to help keep us on track. Hopefully not forget any doses.” [ID 6] It seems like it could help to keep on schedule and just like simple reminders of what to do, certain things especially get put and medications.” [ID 13]	“I think having something to be able to keep track of things for you is the number one way to get to have success with them after care because when I left the hospital, like the first time after he had his treatment, I was completely overwhelmed. It was like, how am I going to keep track of all of this stuff?” [ID 3] “It was nice because if you just didn’t realize what time was, it would ring and kind of let you know like, hey, it’s time to take medications that so just getting into the swing and kind of getting used to taking all that medication too so.” [ID 2]
Helpful	“I think it would be helpful. You know, just having it. To mark off you’ve taken it or having the option to have notes to look back on. Have it all on one place and you always have your phone with you, so.” [ID 2] “Yeah, I mean, especially if she’s taking a lot of meds I don’t remember, like knowing like getting a reminder would probably help you.” [ID 8]	–^a^
Technical difficulties	–	“The other thing I noticed that sometimes it reminded me, sometimes it didn’t.” [ID 3] “It says symptoms recorded and it says what time when the dose was taken at nine p.m. But then you can go back and look, it says it was given at eight p.m.” [ID 1]
Nontech preferences	–	“You got a pencil and the journal, you know I can easily, you know, erase. And I just like to have everything in front of me on one page. I’m a visual person and then like going next to next page, you know?” [ID 5] “I have it in my head, I know what it is to do, so I don’t really need it.” [ID 13]

a Not applicable.

Upon study completion, families reiterated the app’s ease of use and its effectiveness in maintaining medication routines (Table 3). Participants described the app as easy and self-explanatory, allowing them to quickly enter information and interact with it. They emphasized that simplicity was highly desirable, as a complicated interface would deter use. Additionally, participants noted that the app’s ability to help them stay on track with medication regimens was helpful, particularly when life’s demands could interfere with their child’s medication schedule. However, new themes also emerged during the exit interviews. One notable theme was technical difficulties. Some families reported experiencing glitches or issues with the app’s functionality, which occasionally hindered their ability to fully use the app. Another emerging theme was a preference for nondigital methods. A subset of families expressed a preference for traditional, paper-based methods of managing health care routines. These participants indicated that while the app offered useful features, they were more comfortable with paper-based tracking and reminders.

## Discussion

### Principal Findings

The BMT4me app was developed in collaboration with health care providers, caregivers, and patients to aid in medication management, improve adherence monitoring, and track symptoms or medication side effects in real-time [37]. This pilot study aimed to evaluate the usability of the BMT4me app among caregivers of children during the acute phase post-HCT. The caregiver-reported mean SUS score of 73.13 (SD 16.13) at study completion indicated a favorable perception among caregivers, surpassing the threshold of 68 and demonstrating above-average usability. Most caregivers found the app easy to learn and use, with well-integrated features, fostering confidence for independent use at home. These findings are consistent with prior research emphasizing the significance of intuitive design and user-friendly interfaces in mHealth apps [39,40].

The positive usability feedback suggests that the app’s design and features were well-received, enhancing caregivers’ confidence in managing their child’s complex medication regimen [41]. Engagement with the app generally depends on user motivation, perceived value, and satisfaction. As highlighted by Kim et al [42], technology must be both useful and enjoyable, with perceived value and satisfaction stemming from the overall user experience [42]. Caregiver satisfaction emerges as a critical factor for sustainability. Similar to existing literature, out of the participants who use the app regularly, the satisfaction with the BMT4me app was high [43,44]. Most caregivers in this study reported the app as helpful for tracking their child’s medication schedule, finding it easy to use, and expressing willingness to recommend it to others. These sentiments were further echoed in qualitative interviews, with caregivers affirming the app’s ease of use and effectiveness in navigating their child’s post-HCT journey.

Current literature highlights a preference among users for mHealth apps that allow communication with health care providers [45]. This aligns with this study, where 50% (n=7) of caregivers recommended integrating EMR with the BMT4me app and including important contact numbers to facilitate easy communication with health care providers through the app. Including educational content in the mHealth apps has been shown to be preferred among individuals with chronic illnesses [46-48]. This trend was similarly observed in this study, with 14% (n=2) of our participants suggesting the inclusion of information on frequently experienced symptoms after HCT, along with details about medications and their uses.

While initial reactions to the app were positive, the sustainability of long-term use among our sample was constrained. Several caregivers stopped using the app beyond the initial sign-in phase, highlighting potential barriers such as technical challenges, time constraints, feeling overwhelmed, inconvenience, or perceived lack of necessity. Identifying and addressing these barriers is crucial for optimizing app design and implementation strategies to foster sustained engagement among caregivers. Notably, a significant proportion of participants used the app for the first few weeks postdischarge, establishing medication-giving routines before stopping the use. This suggests that the BMT4me app may serve as a valuable resource in assisting families with establishing medication administration habits, particularly during the initial phase of treatment. Some caregivers also expressed the potential value of the BMT4me app in the broader oncology population and wished to have access to it earlier in their child’s treatment journey. The app’s ability to accommodate the transition from a simple to a complex medication regimen, characterized by frequent changes in medications and dosages, underscores its potential utility beyond the HCT context.

Caregiver feedback on the BMT4me app indicated a generally positive experience from enrollment to study completion, however, some areas showed declines. The mean SUS score decreased from 86.15 to 73.13, reflecting a decrease in overall usability satisfaction; nevertheless, it surpassed the 68% cut-off for usability. Initially, 73% (n=11) of caregivers found the app accessible and useful, and 67% (n=10) found it easy to use [31]. These ratings improved with continued interaction, with 88% (n=7) later describing the app as accessible, useful, and valuable. Caregiver satisfaction measure showed minimal change from enrollment to completion, demonstrating high caregiver satisfaction throughout the study.

Analyzing app engagement activities provided insights into caregivers’ usability patterns and preferences. For instance, some features demonstrated more active engagement than others, such that creating and administering doses suggested active engagement in medication management, while note-taking and symptom tracking exhibited low utilization. Therefore, there are potential areas for improvement to better address caregivers’ diverse needs and preferences. This observation aligns with existing literature, which often highlights the importance of user-centered design in health apps to enhance engagement and adherence [49]. Studies have shown that while medication management tools are frequently used due to their direct impact on patient care, features like symptom tracking and note-taking require more intuitive interfaces and clear benefits to encourage consistent use. By addressing these gaps, developers can create more effective and comprehensive health management tools that cater to the varied needs of caregivers, ultimately improving patient outcomes [23,50,51].

Overall, this study underscores the importance of user-centered design with a mixed methods approach. By using multiple methods of data collection and data sources, we gained an in-depth understanding of patients with HCT and their families’ complex needs. The results suggest that while the BMT4me app has significant potential, there are critical areas that require attention for sustained engagement and effectiveness. Future development should focus on overcoming technical challenges, enhancing the perceived value of lesser-used features, and ensuring the app remains a supportive tool throughout the entire treatment journey. By addressing these gaps, the BMT4me app can be optimized to better meet the evolving needs of caregivers and patients and improve their health outcomes and quality of life.

### Limitations

In addition, this study is not without limitations. First, the use of a single pilot recruitment site may restrict the generalizability of our findings. Although we included a diverse group of children who received HCT at this institution, the small number of caregivers may not represent the broader population. The demographic characteristics of our caregiver (female, White, and non-Hispanic) and child (male, White, and non-Hispanic) cohorts are consistent with other studies in this population [52-54]. However, while our cohort reflects the typical demographics of the pediatric HCT population and their caregivers, it is important to acknowledge that our results may be limited in their broader generalizability. Future research should aim to include a more diverse sample to ensure that findings are relevant across different demographic groups. Furthermore, our recruitment was limited to English-speaking caregivers, due to the app only being available in English. Future versions of the app should be developed in multiple languages to allow for more inclusive recruitment and to examine the app’s usability across diverse linguistic and cultural groups. Including caregivers who speak other languages could have enriched the diversity and applicability of our findings. Another limitation is the variability in the timing of app distribution. Participants received the app at different stages of their HCT journey, some on the day of discharge and others after discharge. This inconsistency could have influenced their established medication routines, potentially affecting the outcomes such that participants who were recruited after discharge were less likely to use the app due to already having a system in place for medication management. Additionally, technical issues with the app were noted, which could have impacted the user experience and the app’s usability and sustained engagement. Addressing these barriers is crucial for optimizing the app’s design and ensuring sustained use. Future improvements should focus on enhancing technical stability, expanding user-centered features, and increasing accessibility to diverse populations.

### Conclusions

Despite these limitations, we demonstrated the usability of the BMT4me app among caregivers of children undergoing HCT. This study extends the literature by providing insights into caregiver technology expectations and needs and highlights the potential of mHealth tools to manage medication adherence at home. Notably, 50% (n=7) of caregivers expressed that integrating the app with EMR would be helpful, benefiting both caregivers and health care providers by streamlining access to medication-taking patterns and updating medication regimens. Furthermore, there is potential to adapt this work for children with other chronic conditions, extending the benefits of the BMT4me app beyond the HCT context. The study findings offer valuable insights into the feasibility of conducting a larger randomized controlled trial, potentially leading to significant improvements in adherence and clinical outcomes in children post-HCT.

## Supplementary material

10.2196/66847 Multimedia Appendix 1 Enrollment reaction card responses from caregivers.

10.2196/66847 Multimedia Appendix 2 Exit reaction card responses from caregivers.

10.2196/66847 Multimedia Appendix 3 Frequency of app use across different features.

## Abbreviations

EMR: electronic medical record
HCT: hematopoietic stem cell transplant
mHealth: mobile health
REDCap: Research Electronic Data Capture
SUS: System Usability Scale
