# Supplementary material for: Supporting Medication Adherence in Pediatric Patients Undergoing Hematopoietic Stem Cell Transplant Using the BMT4me mHealth App: Mixed Methods Usability Study
Source: JMIR Cancer. 2025 May 29;11:e66847. doi: 10.2196/66847 (PMC12140368; doi:10.2196/66847)
Supplement: Multimedia Appendix 1 [file cancer-v11-e66847-s001.docx]

**Table 2.** Enrollment reaction card responses from caregivers

| Accessible 11 (73.3%) | Useful  11 (73.3%) | Easy to use  10 (66.7%) | Appealing  8 (53.3%) | Organized  8 (53.3%) | Usable  8 (53.3%) | Valuable  8 (53.3%) |  |
| --- | --- | --- | --- | --- | --- | --- | --- |
| Efficient  7 (46.7%) | Timesaving  7 (46.7%) | Flexible  6 (40.0%) | Straight Forward  6 (40.0%) | Relevant  6 (40.0%) | Connected  5 (33.3%) | Consistent  5 (33.3%) |  |
| High quality  5 (33.3%) | Motivating  5 (33.3%) | Attractive  4 (26.7%) | Customizable  4 (26.7%) | Fast  4 (26.7%) | Comprehensive  3 (20.0%) | Empowering  3 (20.0%) |  |
| Simplistic  3 (20.0%) | Trustworthy  3 (20.0%) | Desirable  2 (13.3%) | Familiar  2 (13.3%) | Reliable  2 (13.3%) | Exciting  1 (6.7%) | Predictable  1 (6.7%) |  |
| Time-consuming  1 (6.7%) | Busy | Collaborative | Complex | Confusing | Fresh | Frustrating |  |
| Fun | Gets in the way | Hard to use | Inconsistent | Intimidating | Inviting | Not valuable |  |
| Overbearing | Overwhelming | Patronizing | Personal | Rigid | Slow | Sophisticated |  |
| Stressful | Stimulating | Too technical | Uncontrollable | Unconventional | Unpredictable |  |  |

>=50% <50% 0%
