# Supplementary material for: Supporting Medication Adherence in Pediatric Patients Undergoing Hematopoietic Stem Cell Transplant Using the BMT4me mHealth App: Mixed Methods Usability Study
Source: JMIR Cancer. 2025 May 29;11:e66847. doi: 10.2196/66847 (PMC12140368; doi:10.2196/66847)
Supplement: Multimedia Appendix 2 [file cancer-v11-e66847-s002.docx]

**Table 3.** Exit reaction card responses from caregivers

| Accessible  7 (87.5%) | Useful  7 (87.5%) | Valuable  7 (87.5%) | Appealing  6 (75%) | Easy to use  6 (75%) | Organized  6 (75%) | Usable  6 (75%) |
| --- | --- | --- | --- | --- | --- | --- |
| Efficient  5 (62.5%) | Flexible  5 (62.5%) | Straight Forward  5 (62.5%) | Time-consuming  5 (62.5%) | Connected  4 (50%) | High quality  4 (50%) | Motivating  4 (50%) |
| Relevant  4 (50%) | Attractive  3 (37.5%) | Comprehensive  3 (37.5%) | Consistent  3 (37.5%) | Customizable  3 (37.5%) | Empowering  3 (37.5%) | Fast  3 (37.5%) |
| Simplistic  3 (37.5%) | Reliable  2 (25%) | Trustworthy  2 (25%) | Desirable  1 (12.5%) | Exciting  1 (12.5%) | Familiar  1 (12.5%) | Predictable  1 (12.5%) |
| Busy | Collaborative | Complex | Confusing | Fresh | Frustrating | Fun |
| Gets in the way | Hard to use | Inconsistent | Intimidating | Inviting | Not valuable | Overbearing |
| Overwhelming | Patronizing | Personal | Rigid | Slow | Sophisticated | Stimulating |
| Stressful | Timesaving | Too technical | Uncontrollable | Unconventional | Unpredictable |  |

>=50% <50% 0%
