# Supplementary material for: Supporting Medication Adherence in Pediatric Patients Undergoing Hematopoietic Stem Cell Transplant Using the BMT4me mHealth App: Mixed Methods Usability Study
Source: JMIR Cancer. 2025 May 29;11:e66847. doi: 10.2196/66847 (PMC12140368; doi:10.2196/66847)
Supplement: Supplementary file 3 [file cancer-v11-e66847-s003.docx]

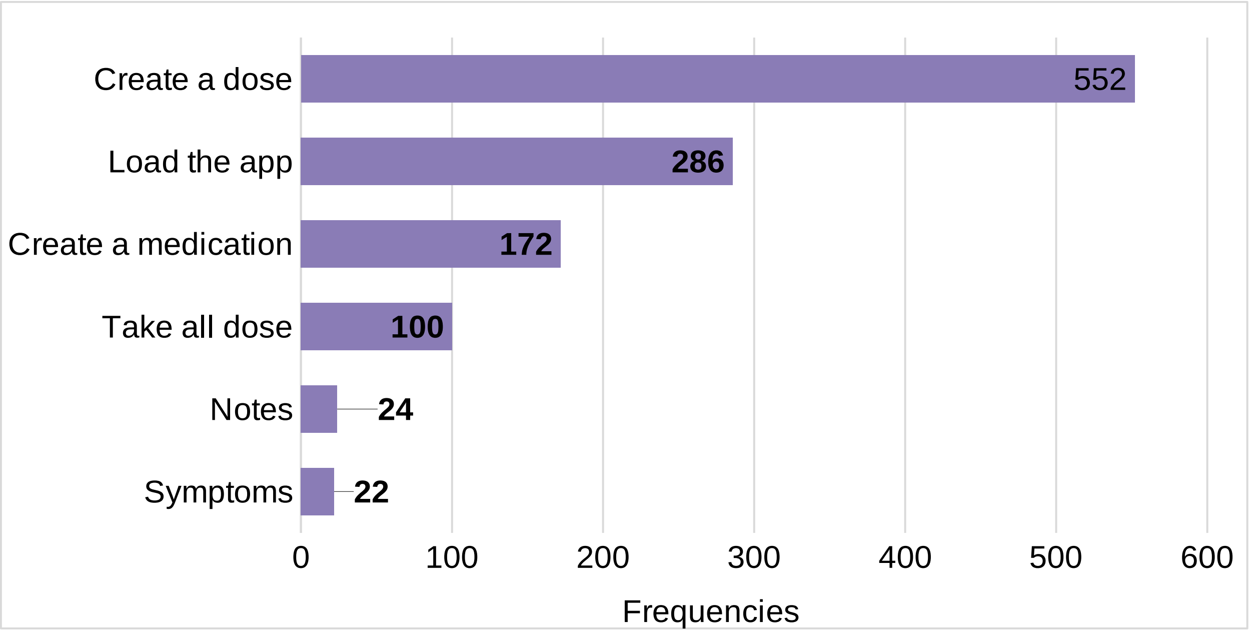
Create a dose
Load the app
Create a medication
Take all dose
Notes
Symptoms
552
286
172
100
24
22
0
100
200
300
400
500
600
Frequencies
